# Determinants of puerperal sepsis among post partum women at public hospitals in west SHOA zone Oromia regional STATE, Ethiopia (institution BASEDCASE control study)

**DOI:** 10.1186/s12884-019-2230-x

**Published:** 2019-03-18

**Authors:** Getu Alemu Demisse, Samuel Dessau Sifer, Buseraseman Kedir, Daniel Belema Fekene, Gizachew Abdissa Bulto

**Affiliations:** 1 Department of Public health, College of Medicine and Health Sciences, Ambo University, Ambo, Ethiopia; 2 Departmentof Nursing, Arbaminch college of health science, Arbainch, Ethiopia; 3 Department of Midwifery, Bona hospital, Bona, Ethiopia; 4 Department of midwifery, Ambo University College of medicine and health science, Ambo, Ethiopia

**Keywords:** Puerperal sepsis, West shoa, Oromia, Ethiopia, Arbaminch, Determinant, Cases, Controls

## Abstract

**Background:**

Puerperal sepsis is an infection of the genital tract, which occurs from rupture of amniotic sacs and within 42^nd^day after delivery. It happens mainly after discharge in the 1st 24 h of parturition. It is the third leading cause of direct maternal mortality in developing nations. It is also among preventable conditions. Even though multiple interventions were done to overcome these health problems, maternal mortality and morbidities were still significant. Mainly, in Ethiopia lack of clearly identified causes of maternal mortality and morbidity makes the problem unsolved.

**Methods:**

Case-control study was conducted at public Hospitals in west shoa zone Oromia regional state, Ethiopia from February 01 to April 30/2018.women with puerperal sepsis (*n* = 67) were selected by convenience method. Controls (*n* = 213) were selected by systematic random sampling. Controls to cases ratio was 3:1 and structured questionnaire was used to interviewafter verbal consent was obtained. Data was entered in to epi –info 7.2 then exported to SPSS version 20.0 for analysis. A logistic regression model was used for data analysis. Those variables which have *p*-value < 0.05 were accepted that they are independent determinants of puerperal sepsis.

**Result:**

Rural residence (AOR [95%CI] = 2.5(1.029–6.054),Mothers with no formal education (AOR [95%CI] = 6.74([1.210–37.541]), up to primary level of education(AOR [95%CI] = 6.72(1.323–34.086), total monthly income of the mother or family<=500 ETB and 501–1500 ETB(AOR [95%CI] = 5.94(1.471–23.93) and (AOR [95%CI] =6.57 (1.338–32.265) respectively, Mothers having 1–2 times antenatal care(ANC)visit (AOR [95%CI] = 6.57([1.338–32.265]), Duration of Labor12–24 h (AOR [95%CI] = 3.12 (1.805–12.115),> = 25 h (AOR [95%CI] = 4.71([1.257–17.687]),vaginal examinations > = 5times (AOR [95%CI] = 4.00([1.330–12.029]), Delivery by C/S (AOR [95%CI] = 3.85 ([1.425–10.413]), Rupture of membrane > 24 h (AOR [95%CI] = 3.73([1.365–10.208]) and those Referred from other health institutions (AOR [95%CI] = 2.53([1.087–5.884],were independent determinants of puerperal sepsis in this study.

**Conclusion:**

Majority of determinants of puerperal sepsis were related with pregnancy and childbirth. Therefore, to tackle a problem of puerperal sepsis all concerning bodies should take measures during prenatal, natal and postnatal period.

**Electronic supplementary material:**

The online version of this article (10.1186/s12884-019-2230-x) contains supplementary material, which is available to authorized users.

## Background

Puerperal sepsis is an infection of genital tract, which occurs at any time from the rupture of membranes or time of labor and up to 42^nd^days from parturition.It accompanied with 2 or more of the following conditionsare present: pelvic pains, high body temperature (that is oral temperature 38.5 °C or above on any occasion, abnormal genital discharge (presence of pus), bad smell or foul odor of discharge, delay in the reduction of the size of the uterus (< than 2 cm / day with in the first Eight days) [1].

World health organization reported about 358,000 maternal deaths occurring during labor and childbirth and 15% were related with puerperal sepsis. Puerperal sepsis is among the preventable conditions in developing and developed nations.It is mainly occurs after discharge in the 1st 24 h of parturition [2].It is ranked as the sixth leading cause of disease burden for women of age 15-44 years, next to depression, HIV/AIDs, tuberculosis, abortion and schizophrenia [3]. As many as 5.2 million, new cases of maternal sepsis are annually occurring and an estimated 62,000 of maternal deaths will result from the condition [3].Even though maternal mortality is slightly decreasing globally, most maternal mortality occurs during childbirth is high [4].

Global risk factors that contribute to infections are caused by poor hygiene practices during delivery and postpartum [4]. This is related to repeated manipulation of patients during delivery, prolonged time of labor or rupture of amniotic sacs, as well as poor sanitary conditions and poor services within health care facilities [4].In Africa, maternal mortality ratio increased by nearly 5% from 2013 to 2015 [5, 6]. It was planned to reduce maternal mortality by 3/4th by the end of 2015 [7]. The majorly strategies drafted to achieve this plan were, ensuring that every birth occurs with the assistance of skilled health personnel, access to emergency obstetric and newborn care,as well access to family planning and effective referral system [7]. However, this is not achieved in Ethiopia by this year. i.e. it was expected to be reduced to 246/100,000 maternal mortality ratio. Even by the year 2015, it is reported that maternal mortality is still 353/100,000 [7] which is still far from the planed strategies. Sustainable development plan (SDP) indicates that, every country should reduce maternal mortality ratio to < 70/100,000 by the year 2030 [8]. Thus to achieve this plan and speed up maternal mortality reduction, it is better to identify cause and contributors to maternal mortality. This study is based on the indicated gaps and lack of a single study conducted in the country in identifying determinants of puerperal sepsis, which contribute in maternal mortality and high fatality of the case makes the study important.

## Methods

### Study area and period

The study was conducted in west shoa zone Oromia regional state Ethiopia from February 01/ to April 30/2018. West Shoa is one of the 18 zones of Oromia Regional State. Ambo is the capital of the zone and it is found at 114 km to the west of Addis Ababa, the capital city of Ethiopia. There are seven public Hospitals in the zone, 96 public health centers, 77 private clinics, 526 health posts providing Service for about 2,150,045 dwellers(west shoa zone health department report 2016, unpublished).

### Study design and population

Facility -based prospective Case- control study design was employed. All postpartum mothers who get service at public hospitals in the zone were our study population.

### Sample size determination

The sample size was estimated using a double population proportion formula. It was based on high proportion of case (83.8) and controls (16.2) [9].Considering a 95% confidence level, 80% power of the test, with 3:1 ratio of controls to cases, the maximum sample size obtained was 258. The final sample size was determined by adding 10% non-response rate, 258 + 26 = 284 study participants.

### Sampling procedure

All hospitals in the zone giving obstetric and gynecologic services were included in the study.

Postpartum mothers admitted with puerperal sepsis were accepted as cases& selected purposively. Those postpartum mothers who were not reported they have puerperal sepsis were taken as controls. Each control selected from the same hospitals for each case with One to three ratio of case to control.

### Data collection procedures and quality assurance

A structured, interview administered questionnaire which was adapted from study conducted in Kenya (Additional file 1) [10] was used to collect data from the study participants. The questionnaire was prepared in English and translated in to local language Afan Oromo bythetranslator, and then translated back to English by a third person to check for consistency.

A pre-test was done on 5% of the total study participants and necessary adjustmentswere made. Fourteen BSC Nurseswere recruited as data collectors and three-health officers were supervisors. Data collectors and supervisors were trained about the study instrument and data collection procedure. The principal investigator and the supervisors checked the collected data for completeness. Theinterview was conducted after the patients treated for puerperal sepsis. Participants who feel discomfort during the interview were given ant pain and even stop the interview for a while until they feel good.

### Data management and analysis

Data was coded, entered in to Epi info version 7.2,and then transported to SPSS version 20.0Itwasrecorded, categorized, sorted, cleaned and analyzed using SPSS. Frequency distribution was done to check missing data. Categorical variables were expressed as frequency and percentages. Cross tabulations of independent variables with the occurrence or experiences of puerperal sepsis were performed. On Logistic regression, Initially, All independent variables were analyzed by bivariate logistic regression analysis. Those variables with p -value < 0.25 in the bivariate analysis were taken and analyzed by back ward stepwise method in multivariable logistic regression. Hosmer-lemeshow test was used for checking model fitness with *p*-value > 0.05.Those variables, with p-value < 0.05, were taken as statistically significant determinants of puerperal sepsis.

### Ethical approval

Ethical review Committee (IRC) of Arbaminch University approved the proposal of this research. Letter of permission was obtained from West shoa Zone health department and from each hospital administrative. Informed verbal consent was obtained from the study subjects after the data collectors explained the study objectives, procedures and their right to refuse not to participate in the study. Furthermore, confidentiality of the study subjects was assured.

## Results

### Demographic and socio-economic characteristics of participants

Overall, the demographic and socio-economic characteristics of the cases and controls were almost similar.Total 280 mothers were participated in the study (67 = cases) and (213 = controls) with anoverall response rate of 99.6%. Results in this study shows that the age of respondents ranged from 16 to 40 with a mean age of 26(±5) years among controls and 24(±4) years among cases. Majority of the participants were within age group of 25–29. From this age group, 22.4% were from cases and 42.7% were from among controls. Majority of the study participants were multiparous, composing about 56.8%. About 60.7% of mothers with puerperal sepsis were delivered spontaneously (Table 1).

**Table 1:** Socio demographic characteristics of respondents in Public hospitals in west shoa zone Oromia, Ethiopia April 2018

Variables	categories	Cases 67 = (%)	Controls 213 = (%)	Total (N%) = 280
Age group	< 20 yrs	16 (23.9)	18 (8.4)	29 (10.4)
	20-24 yrs	4 (6.0)	51 (23.9)	72 (25.7)
	25-29 yrs	15 (22.4)	91 (42.7)	106 (37.9)
	30-34 yrs	21 (31.3)	51 (23.9)	42 (15.0)
	> 34 yrs	11 (16.4)	18 (8.5)	31 (11.1)
Residence	Urban	20 (29.9)	152 (71.4)	172 (61.42)
	Rural	47 (70.1)	61 (28.6)	108 (38.6)
Educational level	Tertiary(college or university	4 (6.0)	79 (37.1)	83 (29.6)
	Secondary	25 (37.3)	50 (23.5)	75 (26.8)
	Primary None /no education	18 (26.9) 20 (29.9)	35 (16.4) 49 (23)	53 (18.9) 69 (24.6)
Monthly income	> 4500	5 (7.5)	27 (12.5)	32 (11.4)
	3501–4500	4 (6.0)	40 (18.8)	44 (15.7)
	2501–3500	10 (14.9)	73 (34.3)	83 (29.9)
	1501–2500	3 (4.5)	41 (19.2)	44 (15.7)
	501–1500	16 (23.9)	13 (6.1)	29 (10.4)
	<=500	29 (43.3)	19 (8.9)	48 (17.1)
Maternal occupation	Employed/government	9 (13.4)	59 (27.7)	68 (24.3)
	House wife	5 (7.5)	17 (8.0)	22 (7.9)
	unemployed	51 (76.1)	131 (61.5)	182 (65.0)
	Others	2 (3.0)	6 (2.8)	8 (2.9)
Marital status	Married	62 (92.5)	209 (98.1)	271 (96.8)
	single	5 (1.9)	5 (7.5)	9 (3.2)
Husbands ‘occupation	Employed/Government	13 (19.4)	80 (37.6)	93 (33.2)
	Merchant	18 (26.9)	102 (47.9)	120 (42.9)
	Farmer	35 (50.7)	25 (11.7)	60 (21.4)
	Other	–	4 (1.9)	4 (1.9)

### Determinants of puerperal sepsis

Residence of mothers was significantly associated with puerperal sepsis. It shows that women who live in the rural areas were 2.5 times more likely to develop puerperal sepsis when compared to those mothers living in the urban area (AOR: 2.5; 95% CI 1.03, 6).

Educational level of mothers is also associated with the puerperal sepsis. Those Mothers who have no formal education affected (AOR: 6.8; 95%CI 1.2, 37.5), and those who completed up to primary level were also developed puerperal sepsis (AOR: 6.7;95% CI 1.3, 34).Furthermore, monthly income is also associated with puerperal sepsis. Mothers who have total monthly income /total family income <=1500 ETB/month were also developed puerperal sepsis compared to those who get total monthly income of > = 4500ETB (AOR: 5.9; 95%CI 1.471, 23.9).

Number of Antenatal care follow up is another significant variable associated with puerperal sepsis. The result shown that mothers who have ANC follow up one to two times were 4.2 times more likely to develop puerperal sepsis compared to those who had three to four times(AOR:4.2;95%CI 1.74,10).

Mothers who were in labour for > = 25 h were 4.7times more likely to develop puerperal sepsis compared with those < 12 h duration on labour (AOR: 4.7; 95% CI 1.26, 17.7).Number of vaginal examination was also identified as determinant of puerperal. Participants who undergo five or more vaginal examination (VE) during delivery were 4.0 times more likely to develop puerperal sepsis (AOR: 4.0; 95%CI 1.3, 12.0). Mode of delivery is also significant determinant of puerperal sepsis in this study. It was indicated that mothers who delivered by cesarean section(c/s) were 3.8 times more likely to develop puerperal sepsis compared to those delivered by spontaneous vaginal delivery (SVD) (AOR: 3.85; 95%CI1.43, 10.4). Participants whose amniotic membrane ruptured 24 h before delivery were 3.7 times more likely to develop puerperal sepsis compared to those < 24 h duration (AOR: 3.7; 95%CI1.37, 10.2). Mothers, who were referred from other health institutions were 2.5.times more likely to develop puerperal sepsis (AOR: 2.5; 95%CI1, 5.9) (Table 2).

**Table 2:** Multivariable logistic regression analysis Result indicating determinants of puerperal sepsis among postpartum mothers in Public Hospitals in West shoa zone Oromia, Ethiopia April 2018

variables	category	Puerperal sepsis		COR(95% C.I)	AOR(95%CI)
		Case = 67(%)	Control = 213(%)		
Residence	Urban	20 (29.9)	152 (71.4)	1	1
	Rural	47 (70.1)	61 (28.6)	5.86 (3.2–10.7)	2.5 (1.029–6.054)
Educational level of mother	Tertiary(college or university	4 (6.0)	79 (37.1)	1	1
	Secondary	25 (37.3)	50 (23.5)	9.88 (3.24–30.06)	6.5(.181–35.53)
	Primary	18 (26.9)	35 (16.4)	10.1 (3.2–32.21)	6.7 (1.32–34.0)
	None /no education	20 (29.9)	49 (23)	8.06 (2.601–24.98)	6.8 (1.21–37.5)
Monthly income of mother/family	> = 4501	5 (7.5)	27 (12.5)	1	1
	3501–4500	4 (6.0)	40 (18.8)	.54 (.133–2.195)	0.38(.06–2.35)
	2501–3500	10 (14.9)	73 (34.3)	.740(.232–2.361)	1.03(.23–4.7)
	1501–2500	3 (4.5)	41 (19.2)	.395(.087–1.91)	0.33(.04–2.6)
	501–1500	16 (68.7)	13 (6.1)	6.65 (1.99–22.12)	5.94 (1.5–23.9)
	< 500	29 (43.3)	19 (8.9)	8.24 (2.7–25.18)	6.57 (0.34–32.3)
Duration of labour	< 12 h	13 (19.4)	71 (33.3)	1	1
	12–24 h	18 (26.9)	103 (48.4)	.95(.44–2.07)	3.12 (0.8–12.12)
	> = 25 h	36 (53.7)	39 (18.3)	5.041 (2.39–11.)	4.75 (1.26–17.7)
Mode of delivery	Spontaneous Vaginal delivery	26 (38.8)	144 (67.6)	1	1
	Instrumental delivery	17 (25.4)	25 (11.7)	3.77 (1.79–7.9)	1.29(.41–4.01)
	Caesarean section	24 (35.8)	44 (20.7)	3.02 (1.58–5.784)	3.85 (1.43–10.4)
Number of ANC follow up	3–4 times	28 (41.8)	156 (73.2)	1	1
	1–2 times	39 (58.2)	57 (26.8)		
Duration of rapture of membrane	<=24 h	45 (67.2)	179 (84.0)	1	1
	> = 25 h	22 (32.8)	34 (16.0)	2.57 (1.37–4.82)	3.73 (1.37–10.2)
Number of vaginal examinations	1–2 times	20 (29.9)	107 (50.2)	1	1
	3–4 times	15 (22.4)	41 (19.2)	0.7(.28–1.75)	2.0 (.65–6.3)
	> = 5times	24 (35.8)	35 (16.4)	3.67 (1.812–7.43)	4.(1.3–12.03)
	Don’t know	8 (11.9)	30 (14.1)	2.57 (1.00–6.56)	2.3(.53–9.7)
Referral status	Yes	30 (44.8)	155 (72.8)	1	1
	No	37 (55.2)	58 (27.2)	3.3 (1.86–5.82)	2.5 (1.09–5.9)

## Discussion

This study tried to identify the determinants of puerperal sepsis in the study area.

In this study, rural dwellers were 2.5 times more likely to develop puerperal sepsis compared with that of mothers from urban. This is in line with a study conducted in Uganda [10]. But Study from Bangladesh contrast with this study, showing that no significant association between puerperal sepsis and place of living [9].The association may be due to unclean delivery at home, low awareness about ANC follow up and low sanitation in rural areas.

In this study mothers with total family with monthly income 501–1500 ETB were 5.94 times more likely to develop puerperal sepsis compared with those who get > = 4500ETB.A study from Bangladesh [9] and Alexandria Egypt(28) evidenced that exposure to lower socio economic status of mothers fall mothers in risk of developing puerperal sepsis. This might be due to that mothers in low socio economic status cannot afford for their health expense, poor nutrition and low immunity in resisting themselves.

Participants who have no formal education were 6.7 times and those learned up to primary level of education were 6.8 times more likely to develop puerperal sepsis compared to those who joined college and above respectively. Similar findings have been revealed that 96% of women were uneducated and only the rest had a primary level of education from those developed puerperal sepsis [9]. A study from Karachi Pakistan [11],Alexandria Egypt (28) also in line with this study.

It was shown that mothers with rapture of membrane > 24 h were 3.7 times More likely to develop puerperal sepsis compare to those have amniotic fluid leakage 24 h or below before delivery as this study indicated. It shows22 (32.8%) of cases with amniotic fluid leakage were developed puerperal sepsis when compare to 34(16.0%) of controls with the leakage. Study from Pakistan revealed that 68 (73.8%) of cases with puerperal sepsis were those who have prolonged history of amniotic liquor(amniotic fluids) leakage 48-72 h before delivery [12]. Study from Kenya [13] and Pakistan [11] also in lined with this study. This is due to prolonged exposure of internal parts when natural protections like membrane is removed and prolonged opening of cervix which leads in entry of ascending microorganisms.

Number of Vaginal examination is also associated with puerperal sepsis in this study, 24(35.8%) of cases had vaginal examination > = 5 times compare to 35(16.4%) of controls > = 5 times vaginal examinations during delivery. Those mothers who had > = 5 times vaginal examination during delivery had 4.0 times more likely to develop puerperal sepsis compared to those who had Vaginal examination 1–2 times. This is in a row with study conducted in Egypt which indicated that having > = 5 times vaginal examination can lead in developing puerperal sepsis [14, 15].Similarly systemic review study conducted in south Asia evidenced that putting hands, frequently in vagina end with puerperal sepsis [16]. Study from Kenya reported that women who had 2or more vaginal examination were 3.95 times more likely to develop puerperal sepsis [17].This frequent manipulation of genital tracts will facilitate ascension of microorganisms from lower genital tract and thereby increase in probability to develop puerperal sepsis.

The study result also indicated that mode of delivery is significantly associated with puerperal sepsis.

That is mothers who delivered by caesarean section were 3.9 times more likely to develop puerperal sepsis when compared to those delivered by spontaneous vaginal delivery. Studies from different regions [18],42 [19]) were in line with this study .

In this study mothers who was in labor for 12–24 h& > 25 h were 3.1 &4.7times more likely to develop puerperal sepsis compared to those in labor < 12 h respectively. This is in line with study conducted in Kenya which exposed that mothers in labor for > 25 h were 3.95times more likely at risk in developing puerperal sepsis [17].Studies from Nepal [12], patan hospital Nepal [2] and Tanzania summarizes duration of labor contribute in the development of puerperal sepsis as prolonged labor with frequent vaginal examinations leads to puerperal sepsis.

This study also revealed number of ANC follow up is significantly associated with puerperal sepsis. Those mothers who had ANC follow up one to two times be 4.2times more likely to develop puerperal sepsis when compared to those who had three to four times. This is supported by study from Pakistan.

Referral status of mothers was also independent determinant of associated with puerperal sepsis in this study. Those mothers who referred from other health institutions were 2.5 times more likely to develop this health problem. Study from Pakistan in line with this result in which (73.64%) of cases were referred from outside of the study hospital [11].Study from Uganda also in line with this study in which cases referred from other health institutions were more likely to develop puerperal sepsis when compared to that those from study hospital [10]. This might be due to prolonged time to arrive the hospital and probable unclean vaginal examination on the way to hospital will contribute in development of puerperal sepsis.

### Limitation

Because the study conducted only at public hospitals, those mothers who went to private clinic my missed and they can be different in socio economic status from those treated at public hospitals.

## Conclusion and recommendation

This study revealed majority of puerperal sepsis occurs during Natal and postnatal period in general. Rural residence, Educational level of primary or less, monthly income of mother or family <=1500ETB, Being on labour for > 24 h, delivered by caesarean section, Having ANC follow up to two or less, ruptured membrane> 24 h before delivery, frequent vaginal examination, and being referred from other health institution were identified determinants of puerperal sepsis. Considering this, community awareness about frequency of ANC, cares needed during post partum, scaling up educational level of community and supporting those in low socio economic status were recommended interventions.

## Additional file

Additional file 1: Questioners. (DOCX 19 kb)

## Abbreviations

AIDS: Acquired immune deficiency syndrome
ANC: Antenatal care
AOR: Adjusted odds ratio
BSC: Bachelor of science
C/S: Caesarean section
COR: Crude odds ratio
ETB: Ethiopian birr
HIV: Human immune virus
HO: Health officer
PS: Puerperal sepsis
SDG: Sustainable development goal
SPSS: Statistical package for social science
SVD: Spontaneous vaginal delivery
