# Supplementary material for: Determinants of puerperal sepsis among post partum women at public hospitals in west SHOA zone Oromia regional STATE, Ethiopia (institution BASEDCASE control study)
Source: BMC Pregnancy Childbirth. 2019 Mar 18;19:95. doi: 10.1186/s12884-019-2230-x (PMC6423770; doi:10.1186/s12884-019-2230-x)
Supplement: Supplementary file 1 — Questioners. (DOCX 19 kb) [file 12884_2019_2230_MOESM1_ESM.docx]

**ADDITIONAL FILES**

1. **Questioners**

This questionnaire is meant to collect information about you and your experience as our patient. The questionnaire gives important feedback, which will be used to increase the provision of a better health service care and Help in prevention of the problem of puerperal sepsis. Your responses will be treated with utmost confidentiality. Respond kindly to all items with honesty. Thank you for your cooperation.

### (I): DEMOGRAPHIC AND SOCIO-ECONOMIC CHARACTERISTICS

Table 1.Questioner for post partum mothers

| code | question | response | | skip |
| --- | --- | --- | --- | --- |
| 001 | What is your age? | --------------- | |  |
| 002 | Where did you live | 1. Rural 2. urban | |  |
| 003 | What level of education did you complete? | 1] None [2] primary [3] secondary  [4] tertiary/college | |  |
| 004 | What is your occupation? | [1] House wife [2] self employed  [3] Employed [4] others /specify)…… | |  |
| 005 | What is your marital status? | [1] Single [2] married  [3] Others,(specify)…………………… | |  |
| 006 | What is your husbands’ occupation? | ----------------- | |  |
| 007 | How many deliveries have you had? | [1] 1 [2]2 [3]3 [4] ≥4 | |  |
| **(II), RISK FACTORS/OBSTETRIC PROFILE** | | | | |
| 008 | Where do you normally have your deliveries done? | | [1] Home  [2] Health care facility  [3] Tertiary care level  [4]Others,(specify)…………… |  |
| 009 | Did you have multiple pregnancies? if no skip to 011 | | [1] Yes  [2] No | skip |
| 010 | If yes for question No7 how many times | | [1] 1 [2] 2 [3] 3  [4] >=4 |  |
| 011 | What was the mode of your last delivery? | | [1] Spontaneous vaginal delivery  [2] caesarean delivery  [3] instrumental/assisted delivery |  |
| 012 | What was the duration of labour in your last delivery? | | [1] 6-12 hrs  [2] 12-24 hrs  [3] 24-36 hrs |  |
| 013 | How did your labour start? | | [1] spontaneous [2] induction  [3] other---- |  |
| 014 | How long did the rapture of the membrane take? | | [1] ≥24 hrs [2] ≥ 36hrs  [3] ≥48 hrs [4] ≥72hrs |  |
| 015 | Were you assisted during delivery? If no skip to 018 | | [1] Yes [2] No (if No, Proceed to question 8 | skip |
| 016 | For Qu.015, If Yes, Who assisted you? | | [1] Family member  [2] Traditional birth attendant  [3] Doctor [4] lady health worker |  |
| 017 | b). What kind of hygienic practice did you observe from the person assisting you.(tick any that you observed) | | [1]. Washed hands before assisting.  [2]. Used cloves  [3] None [4] Don‘t know |  |
| 018 | Have you developed any of the following symptoms in the last 72 hours (tick all that you have ever suffered from?) | | [1]. Abnormal vaginal discharge  [2]. Pelvic pain  [3]. fever  [4]. Abnormal smell/foul odor of discharge  [5]. Delay in the size of uterus  [6]. Never suffered from any |  |
| 019 | If any of the above, did you seek treatment or help? If no skip to 021 | | [1]. Yes [2]. No | skip |
| 020 | If yes, from who? | | [1]. A government Health center  [2]. Self-treatment  [3]. community member  [4]. Relative/sister/brother  [5]. Private Clinic/Drug Shop/Pharmacy [6]. Herbalist |  |
| 021 | Did you go for antennal care before delivery? | | [1]Yes [2] No |  |
| 022 | If yes for question 021 at which week? | | [1] <12 week [2] –12- 16 wk  [3] >16-20 week [4] >20wk |  |
| 023 | If No for Qu.021, why? | | [1] No money to pay for services [2] Not aware of such services [3] religious issues  [4] Cultural beliefs/taboos  [5] Hospitals are far [6] No vehicles [7] Fear, other, specify……. |  |
| 024 | Have you been diagnosed of any disease before delivery? If no skip to 025 | | [1] No [2] Yes |  |
| 025 | if yes, specify………. | | …………….. |  |
| 026 | How many vaginal examinations did you have before delivery? | | [1] One [2] two  [3] Several [4] none [5] don‘t know |  |
